# A Comprehensive Review on Platelet-Rich Plasma Activation: A Key Player in Accelerating Skin Wound Healing

**DOI:** 10.7759/cureus.48943

**Published:** 2023-11-17

**Authors:** Hardik Patel, Aditya Pundkar, Sandeep Shrivastava, Rohan Chandanwale, Ankit M Jaiswal

**Affiliations:** 1 Orthopedics, Jawaharlal Nehru Medical College, Datta Meghe Institute of Higher Education & Research, Wardha, IND; 2 Orthopedic Surgery, Jawaharlal Nehru Medical College, Datta Meghe Institute of Higher Education & Research, Wardha, IND

**Keywords:** regenerative medicine, safety concerns, clinical applications, activation methods, wound healing, platelet-rich plasma (prp)

## Abstract

Platelet-rich plasma (PRP) activation is emerging as a promising and multifaceted tool for accelerating skin wound healing. This review extensively examines PRP's role in wound healing, focusing on its composition, mechanisms of action, activation methods, and clinical applications. PRP's potential to enhance both chronic and acute wound healing and its applications in cosmetic and aesthetic procedures are explored. Furthermore, this review investigates safety concerns, including adverse reactions, infection risks, and long-term safety implications. Looking to the future, emerging technologies, combination therapies, personalized medicine approaches, and regulatory developments are discussed, pointing towards an important and transformative era in wound healing and regenerative medicine. With its wide-ranging implications for healthcare, PRP activation has the potential to become a ubiquitous and essential therapeutic option, improving patient outcomes and reducing healthcare costs.

## Introduction and background

Wound healing is a complex and dynamic process that plays a crucial role in the body's ability to repair damaged tissue and maintain its structural and functional integrity. The body's natural healing mechanisms come into play when the skin is injured through trauma, surgery, or various medical conditions. Skin wound healing is exciting due to its widespread prevalence, impacting millions of individuals worldwide. Understanding the intricacies of this process is essential for improving the quality of healthcare and the overall well-being of patients [1].

The rate at which a skin wound heals plays a crucial role in a patient's recovery, overall health, and quality of life. Prolonged wound healing can give rise to complications such as infection, scarring, chronic pain, and reduced mobility. Therefore, the medical community has a sustained interest in strategies to enhance the wound-healing process. Facilitating an improved wound-healing process reduces the risk of complications and alleviates the economic burden on healthcare systems and patients [2].

Platelet-rich plasma (PRP) has emerged as a promising therapeutic option in wound healing. PRP is a blood component that contains a concentrated mixture of platelets, growth factors, and bioactive molecules. These components play critical roles in the body's natural response to injury and can enhance the wound-healing process. PRP therapy is particularly appealing because it can be obtained from the patient's blood, reducing the risk of adverse reactions and compatibility issues [3].

This comprehensive review aims to delve into the role of PRP activation in enhancing skin wound healing. We will explore the composition of PRP, its mechanisms of action, and the various methods used to activate PRP. Additionally, we will examine the clinical applications of activated PRP in managing chronic wounds, acute injuries, and cosmetic/aesthetic procedures. Safety concerns and potential side effects will also be addressed. Furthermore, we will discuss the challenges and limitations associated with PRP therapy, including variability in PRP composition, standardization issues, and cost considerations. Lastly, we will highlight the future directions and research trends in this exciting field, which have the potential to reshape the way we approach wound healing and regenerative medicine.

## Review

Mechanisms of PRP action in wound healing

Platelet Function in Wound Healing

Platelets are small, disc-shaped blood cells that play a crucial role in the initial phase of wound healing, known as hemostasis. When a blood vessel is injured, platelets are swiftly recruited to the injury site. Upon reaching the injured area, they undergo a process of activation, where they change shape and release various bioactive molecules stored within their granules. This process leads to the formation of a hemostatic plug, which helps to staunch bleeding and prevent further blood loss [4]. The role of platelets in wound healing extends beyond their function in hemostasis. Once activated, platelets release many bioactive molecules, including growth factors, cytokines, and chemokines, that play pivotal roles in the subsequent phases of wound healing. These bioactive molecules contribute to the recruitment and activation of various cell types involved in the wound-healing cascade, such as immune cells, fibroblasts, and endothelial cells [5].

The released growth factors, including platelet-derived growth factor (PDGF), transforming growth factor-beta (TGF-β), and vascular endothelial growth factor (VEGF), promote cell proliferation, angiogenesis, and the synthesis of extracellular matrix components, all of which are critical for tissue repair and regeneration. Additionally, platelets release factors that modulate the inflammatory response, promoting a balanced and controlled immune reaction necessary for clearing debris and pathogens from the wound site [6]. Platelets serve as key orchestrators of the intricate wound-healing process through these mechanisms, initiating the cascade of events that lead to inflammation, cell proliferation, and tissue remodeling. Their role in releasing bioactive molecules and their contribution to regulating the wound-healing microenvironment highlight their significance in healing [7].

Role of Growth Factors and Cytokines

Platelet-rich plasma (PRP) is a concentrated source of growth factors and cytokines, including but not limited to platelet-derived growth factor (PDGF), transforming growth factor-beta (TGF-β), vascular endothelial growth factor (VEGF), and epidermal growth factor (EGF). These bioactive molecules are pivotal in regulating and coordinating the complex cellular activities involved in wound healing and tissue regeneration [8]. PDGF is crucial in wound healing by promoting cell proliferation and migration, particularly of fibroblasts and smooth muscle cells. This synthesizes extracellular matrix components like collagen and fibronectin, which are essential for tissue repair and scar formation. Additionally, PDGF contributes to angiogenesis, which is the formation of new blood vessels, ensuring the injured tissue receives an adequate blood supply for proper healing [9].

TGF-β is involved in several critical aspects of wound healing, including inflammation, cell differentiation, and the synthesis of extracellular matrix proteins. It acts as a potent immunomodulator, helping to regulate the inflammatory response to the injury. Moreover, TGF-β stimulates the differentiation of fibroblasts into myofibroblasts, which are crucial for wound contraction and tissue remodeling [10]. VEGF plays a central role in angiogenesis by stimulating the growth of new blood vessels, a process essential for delivering oxygen and nutrients to the healing tissue. Improved vascularization ensures a more efficient wound-healing process [11].

EGF, on the other hand, plays a pivotal role in promoting epithelial cell growth and differentiation, which is particularly important for the regeneration of the outermost layer of the skin. This is vital in skin injuries, burns, or other conditions where the skin's barrier function is compromised [12]. These growth factors and cytokines within PRP orchestrate the intricate cellular responses required for efficient wound healing by serving as signaling molecules. They stimulate cell migration, proliferation, differentiation, and extracellular matrix production, thereby accelerating tissue repair, reducing scar formation, and expediting wound healing. PRP is a promising therapeutic approach for various wound types and regenerative medicine applications [13].

Immunomodulatory Effects of PRP

Platelet-rich plasma (PRP) contributes to tissue repair and regeneration and possesses significant immunomodulatory effects that are pivotal in orchestrating the immune response during wound healing. These effects are essential for maintaining a balanced and controlled inflammatory environment for effective and uneventful wound healing [14]. PRP has been demonstrated to modulate the activity of various immune cells involved in the wound-healing cascade, such as macrophages, neutrophils, and lymphocytes. Macrophages, in particular, are central players in the immune response, responsible for clearing debris, pathogens, and apoptotic cells from the wound site. PRP influences macrophages to assume an anti-inflammatory or M2 phenotype associated with tissue repair and regeneration rather than a pro-inflammatory response. This polarization of macrophages by PRP promotes a healing environment, reducing inflammation and minimizing the risk of chronic inflammation at the wound site [15].

Neutrophils, a pivotal immune system component, play a crucial role in the initial defense against pathogens. However, their activity must be finely regulated to prevent potential tissue damage. The application of PRP contributes to this regulation by modulating specific neutrophil activities. This ensures that neutrophils effectively clear pathogens while mitigating any excessive responses that could lead to collateral damage to healthy tissue [16]. Additionally, PRP's immunomodulatory effects extend to lymphocytes, aiding in regulating the adaptive immune response. This dual action helps prevent prolonged inflammation and tissue damage by avoiding excessive immune reactions [3].

Angiogenesis and Tissue Regeneration

Platelet-rich plasma (PRP) plays a crucial role in wound healing by promoting angiogenesis, a process that involves the formation of new blood vessels. This mechanism is vital for ensuring the successful regeneration of injured tissues and restoring their structural and functional integrity [17]. PRP contains a rich assortment of growth factors, including vascular endothelial growth factor (VEGF), which is instrumental in stimulating the proliferation and migration of endothelial cells. Endothelial cells are the building blocks of blood vessels, and when VEGF prompts them, they begin to form new vascular structures. This neovascularization serves several vital functions during the wound-healing process [18].

First and foremost, the new blood vessels improve the delivery of oxygen and essential nutrients to the injured tissue. Adequate oxygen and nutrient supply are critical for the cells involved in tissue repair, enabling them to function optimally and expedite the healing process. This improved blood flow also helps remove waste products and metabolic byproducts from the injury site, further supporting regeneration [19]. Additionally, the newly formed blood vessels facilitate the migration of reparative cells, such as fibroblasts, essential for synthesizing extracellular matrix components like collagen. These structural proteins are necessary to restore tissue integrity and heal damaged skin [20].

Activation of PRP

Methods for Activating PRP

Calcium chloride activation: One of the most common methods involves using calcium chloride as an activator. This agent induces a rapid coagulation response in the PRP, causing platelets to degranulate and release their growth factors. Calcium chloride activation is simple, cost-effective, and widely accessible. It is particularly suitable for clinical applications requiring quick and uncomplicated activation [21].

Thrombin activation: Thrombin is another widely used activator for PRP. Thrombin initiates the clotting cascade, leading to platelet degranulation and the release of growth factors. This method is effective and has been utilized in various medical procedures. However, concerns about the potential for thrombin-induced coagulation disorders exist, especially in patients with clotting issues [22].

Exogenous energy sources (ultrasound, laser): In recent years, emerging technologies such as ultrasound and laser have been employed to activate PRP. These methods use mechanical or thermal energy to stimulate platelet degranulation. They offer precise control over the activation process and can be advantageous in clinical situations requiring fine-tuned regulation. However, the equipment and expertise necessary for ultrasound or laser activation can make these methods less accessible [23].

Importance of Activation in Enhancing Wound Healing

Stimulation of cell proliferation: Activated PRP contains growth factors that promote the proliferation of various cell types critical to wound healing. Fibroblasts, for instance, are essential for synthesizing the extracellular matrix components, such as collagen, necessary for tissue repair and scar formation. By stimulating fibroblast proliferation, activated PRP accelerates the deposition of these structural proteins [24].

Facilitation of angiogenesis: Growth factors within activated PRP, such as vascular endothelial growth factor (VEGF), are potent inducers of angiogenesis- the formation of new blood vessels. These newly formed vessels provide the injured tissue with oxygen and nutrients essential for efficient healing. Improved vascularization enhances the delivery of growth factors and immune cells to the wound site, promoting tissue repair [25].

Recruitment of reparative cells: Activated PRP's bioactive molecules act as chemoattractants, drawing in various reparative cells involved in the wound healing cascade. This recruitment includes endothelial cells, fibroblasts, and macrophages. Endothelial cells are instrumental in building new blood vessels, while fibroblasts assist in tissue reconstruction. In their anti-inflammatory M2 phenotype, macrophages support the resolution of inflammation and tissue regeneration [26].

Orchestrated cellular response: The coordinated release of growth factors and cytokines creates an orchestrated response among different cell types. This harmony is crucial for preventing excessive inflammation and scar formation while promoting the effective regeneration of damaged tissue. The balanced interaction of these cells is vital for achieving optimal wound closure and minimizing complications [27].

Comparison of Different Activation Techniques

Speed of activation: Some methods, such as calcium chloride activation, are known for their rapid activation process. This can be advantageous when immediate treatment is required, such as emergency wound care. In contrast, other methods may have a slower activation time, which may be more appropriate for elective procedures or non-urgent applications [28].

Control over growth factor release: The degree of control over the release of growth factors can vary among activation methods. Thrombin activation, for instance, provides precise control, making it suitable for applications where finely tuned regulation of growth factor release is essential. In contrast, methods like calcium chloride may result in a more immediate but less controlled release [29].

Equipment and expertise requirements: Some activation methods, such as ultrasound or laser, require specialized equipment and expertise. These techniques offer precise control but may be less accessible in specific clinical settings due to equipment costs and training requirements. More straightforward methods, like calcium chloride activation, are more readily available and require minimal training [30].

Risk of adverse effects: Considerations of safety are paramount. Some activation methods, such as thrombin-induced clotting, may carry a higher risk of adverse effects. Clinicians need to balance the potential benefits of a method with the associated risks, considering the patient's medical history and specific needs [31].

Clinical application: The choice of activation method should align with the specific clinical application. For example, methods that allow fine control may be more suitable for cosmetic and aesthetic procedures, while rapid activation techniques may be preferred for managing acute wounds [32].

Cost and accessibility: The costs associated with different activation methods should be considered, as well as their accessibility in a given healthcare setting. Cost-effective and widely available methods may be more practical for routine clinical use [33].

Clinical applications of activated PRP in skin wound healing

Chronic Wound Management

Chronic wounds, such as diabetic ulcers, venous stasis ulcers, and pressure sores, represent a substantial healthcare challenge due to their slow healing rates and the potential for severe complications. Traditional wound care must often address chronic wounds' complex and prolonged healing processes. However, activated platelet-rich plasma (PRP) has emerged as a promising therapeutic approach to managing these challenging cases. This section will delve into the clinical applications of activated PRP in chronic wound management, exploring its therapeutic effects, patient outcomes, and the pivotal role it plays in promoting wound closure and tissue regeneration [34].

Activated PRP therapy offers a multifaceted approach to chronic wound management. Its rich concentration of growth factors, including platelet-derived growth factor (PDGF) and transforming growth factor-beta (TGF-β), stimulates cell proliferation, angiogenesis, and the synthesis of extracellular matrix components. These mechanisms are particularly beneficial for chronic wounds, as they facilitate the formation of new blood vessels, enhance tissue repair, and foster the regeneration of damaged skin [14].

Clinical outcomes in chronic wound management with activated PRP have shown promise. Patients with non-healing wounds, often resistant to conventional treatments, have experienced improved wound closure rates and reduced infection rates when PRP therapy is integrated into their care. Moreover, PRP's immunomodulatory effects, such as promoting anti-inflammatory environments, are crucial in mitigating chronic inflammation and tissue damage [35].

The use of activated PRP in chronic wound care accelerates the healing process and improves the quality of life for affected individuals. By promoting effective tissue regeneration and reducing the risk of complications, activated PRP offers hope for patients with chronic wounds who face prolonged suffering and limited treatment options. It has the potential to revolutionize the approach to chronic wound management and set new standards in regenerative medicine, providing patients with renewed prospects for recovery and enhanced well-being [36].

*Acute Wound Healin*g

In acute wound healing, which encompasses surgical incisions, traumatic injuries, and burns, the rapid initiation of the healing process is paramount. Prompt and effective wound closure minimizes complications and plays a pivotal role in achieving optimal patient outcomes. In this context, activated platelet-rich plasma (PRP) has emerged as a valuable tool to enhance acute wound healing by expediting tissue repair, reducing inflammation, and minimizing scar formation [37]. Activated PRP therapy offers a multifaceted approach to acute wound management. Its concentrated growth factors, such as platelet-derived growth factor (PDGF) and transforming growth factor-beta (TGF-β), stimulate various cellular processes critical for wound healing. This includes the promotion of cell proliferation, collagen synthesis, and the formation of new blood vessels (angiogenesis), all of which are essential for rapid tissue repair [17].

Clinical evidence is accumulating to support the use of activated PRP in acute wound healing. Patients undergoing surgeries, experiencing traumatic injuries, or sustaining burns have benefited from the integration of PRP therapy into their treatment regimens. Studies have shown that PRP can lead to faster wound closure, reduced inflammation, and improved overall wound-healing outcomes [38]. Furthermore, activated PRP's role in reducing scar formation is particularly interesting. By promoting efficient tissue repair and reducing inflammation, PRP can contribute to developing less noticeable scars and improving cosmetic and functional outcomes for patients [39].

Cosmetic and Aesthetic Applications

Platelet-rich plasma (PRP) has earned recognition and popularity within the cosmetic and aesthetic industry for its potential to rejuvenate the skin, reduce wrinkles, and enhance overall skin texture. In this context, activated PRP is utilized in various procedures, including PRP facelifts, vampire facials, and hair restoration treatments. This section will delve into the clinical applications of activated PRP in aesthetics, shedding light on the mechanisms that underlie its skin-enhancing effects and showcasing the outcomes achieved through various cosmetic procedures [40]. Activated PRP, when employed in cosmetic and aesthetic treatments, capitalizes on its rich concentration of growth factors, such as platelet-derived growth factor (PDGF) and epidermal growth factor (EGF). These growth factors are instrumental in stimulating collagen production, increasing skin elasticity, and promoting the regeneration of damaged skin cells. By stimulating cell proliferation and angiogenesis, PRP contributes to skin rejuvenation and overall improvement in skin quality [41].

In PRP facelifts and vampire facials, activated PRP is applied topically or injected into the skin. This enhances collagen production, minimizes fine lines and wrinkles, and helps to achieve a youthful and radiant complexion. The minimally invasive nature of these procedures makes them attractive options for those seeking non-surgical cosmetic enhancements [42]. In hair restoration, activated PRP stimulates hair follicles and promotes hair growth. Applying PRP to the scalp fosters the regeneration of hair follicles and increases hair thickness and density. This makes PRP appealing to individuals dealing with hair loss and thinning [43]. The outcomes achieved through activated PRP in cosmetic and aesthetic procedures have been promising. Patients often report improved skin texture, reduced signs of aging, and enhanced hair growth. The natural and regenerative properties of PRP contribute to the appeal of these treatments, as they minimize the risks associated with more invasive cosmetic procedures [44].

Safety and potential side effects

Adverse Reactions and Contraindications

Localized adverse reactions: Following PRP therapy, it is not uncommon for patients to experience localized adverse reactions at the injection site. These reactions typically manifest as pain, swelling, and bruising and are generally transient, subsiding within a few days. Pain management can often be achieved with over-the-counter pain relievers, while cold compresses may help alleviate swelling. Healthcare providers should educate patients about the expected duration and management of these localized adverse reactions, promoting comfort and peace of mind post-treatment [45].

Allergic responses: Although PRP is derived from the patient's blood, there remains a minimal risk of allergic responses, albeit rare. Allergic reactions can occur if a patient exhibits hypersensitivity to any components within the PRP or the materials used in the preparation process. Careful patient screening for known allergies or sensitivities is imperative to mitigate this risk before initiating PRP therapy. This screening process helps identify individuals at higher risk for allergic responses. It allows healthcare providers to take appropriate precautions, such as modifying the PRP preparation process or considering alternative treatment options for these patients [45].

Infection risks: PRP therapy, especially when administered through injections, carries a potential risk of infection. Maintaining strict aseptic techniques and upholding sterility during PRP preparation and administration is essential to minimizing this risk. Infections can lead to more severe complications and should be managed with prompt and appropriate medical care. Patients should also be educated on the signs and symptoms of infection so they can promptly report any concerns to their healthcare providers, allowing for early intervention and treatment if necessary [46].

Contraindications: PRP therapy may not be suitable for certain individuals due to specific contraindications. Contraindications can include patients with bleeding disorders, individuals taking anticoagulant medications, and those with active infections or malignancies. Additionally, individuals with certain medical conditions, such as autoimmune diseases or those who are pregnant, may not be ideal candidates for PRP therapy. A thorough medical evaluation is necessary to determine each patient's eligibility, considering their medical history, current health status, and potential contraindications. Clear communication with patients about their eligibility for PRP therapy is essential to making informed and safe treatment decisions [47].

Infection Risks and Sterility Concerns

Sterile equipment and environments: Ensuring the sterility of all equipment and environments involved in PRP therapy is a foundational requirement. This encompasses using sterile equipment, including centrifuges, syringes, needles, and containers for PRP. Sterilization protocols must be meticulously followed, and all equipment should be regularly maintained and quality-controlled to guarantee both sterility and functionality. This rigorous commitment to sterility prevents contamination and infections during PRP preparation and administration [23].

Aseptic techniques: Healthcare providers conducting PRP therapy must adhere to stringent aseptic techniques to minimize the risk of contamination. Proper handwashing and the use of sterile gloves are critical. Additionally, creating a sterile field, often achieved using sterile drapes and barriers, is essential during the procedure. Any breaches in asepsis, no matter how minor, should be promptly addressed to maintain the integrity of the sterile environment. Aseptic techniques are a barrier against potential sources of contamination and infection [48].

PRP handling: PRP, being a biological product, is particularly vulnerable to contamination. Its handling should occur in a controlled environment, such as a laminar flow hood or a sterile workspace, to minimize the risk of contamination. All materials used for PRP preparation and administration, including tubes, containers, and syringes, should be sterile and single-use to reduce infection risk further [49].

Monitoring and quality assurance: Routine monitoring of equipment, protocols, and PRP preparations is essential to maintaining quality and sterility. Quality control measures should include regular equipment maintenance to ensure proper functioning, routine spore testing of sterilization equipment to verify its effectiveness, and validation of PRP processing techniques to ensure consistent and reliable results. These measures uphold the sterility and quality of PRP preparations and the safety of patients [50].

Patient screening: Before PRP therapy, patients must undergo a thorough screening to identify any pre-existing infections, allergies, or medical conditions that may increase the risk of infection. Patients with active infections or a history of adverse reactions to PRP components may not be suitable candidates for PRP therapy. The screening process is critical to ensuring patient safety and minimizing the risk of adverse events related to PRP treatment [51].

Proper post-treatment care: In addition to maintaining sterility during PRP therapy, healthcare providers should educate patients on proper post-treatment care. This includes providing clear instructions for wound care and emphasizing vigilance for any signs of infection, such as increased pain, swelling, redness, or discharge. Proper post-treatment care ensures that patients are actively involved in their recovery and can promptly report any issues to their healthcare providers, facilitating the early detection and management of potential complications [23].

Long-Term Safety and Potential Risks

Risk of tissue hyperplasia: Prolonged or repeated use of PRP may carry the potential risk of tissue hyperplasia, characterized by the excessive growth and proliferation of cells in response to sustained exposure to growth factors. This phenomenon can result in abnormal tissue growth and has raised concerns about the overstimulation of tissues. Healthcare providers should monitor patients for signs of hyperplasia, particularly in cases of long-term PRP therapy. Ensuring appropriate treatment intervals and conducting regular assessments of tissue response are critical to mitigating this risk [52].

Potential for increased alloimmunity: A notable concern in the context of PRP usage, particularly for individuals undergoing repeated treatments over an extended duration, is the potential elevation in alloimmune responses. While PRP is sourced from the patient's blood, chronic exposure to PRP may heighten the risk of immunological reactions. Despite its autologous origin, the possibility of hypersensitivity reactions or immunological responses to specific components within PRP exists. Vigilant monitoring by healthcare providers is essential to promptly identify any signs of allergic reactions, such as skin rashes or systemic hypersensitivity. Should such reactions be observed, it is imperative to adapt treatment plans to prioritize patient safety [53].

Chronic exposure to growth factors: The long-term effects of chronic exposure to growth factors present in PRP still need to be fully understood. Concerns exist regarding the potential for continuous growth factor exposure to contribute to unintended consequences, such as tissue fibrosis or uncontrolled cell proliferation. Ongoing research is essential to elucidate these potential risks better and establish safe and effective protocols for long-term PRP therapy. Additionally, healthcare providers should consider balancing the regenerative benefits and potential risks when developing long-term treatment plans, ensuring that the advantages outweigh the potential drawbacks [8].

Patient-specific considerations: Tailoring PRP therapy to each patient's needs is paramount, especially when considering long-term use. This individualized approach thoroughly assesses the patient's medical history, underlying health conditions, and response to previous PRP treatments. Patient-specific considerations help healthcare providers determine the appropriate frequency and duration of PRP therapy, aligning the treatment with the patient's specific requirements and minimizing potential risks [54].

Evidence-based practice: Using PRP in clinical settings should be guided by evidence-based practice. While PRP holds significant promise in regenerative medicine, the evolving nature of medical research requires healthcare providers to remain up-to-date with the latest evidence and adhere to best practices. Responsible medical practice involves regularly reviewing and incorporating the most current research findings, treatment guidelines, and safety measures to ensure that PRP therapy is effective and safe for patients. Evidence-based practice promotes the responsible use of PRP in a constantly evolving healthcare landscape where patient well-being remains the top priority [55].

Challenges and limitations

Variability in PRP Composition

Choice of PRP preparation method: The composition and therapeutic potential of PRP are largely dependent on the PRP preparation method. Various preparation techniques yield PRP with differing platelet concentrations and growth factor profiles, including double-spin centrifugation, single-spin centrifugation, and buffy coat preparation. This diversity in methodologies can lead to consistency in treatment responses. For example, the platelet concentration in PRP can vary widely between these methods, impacting the bioactive molecule content and, consequently, the regenerative capabilities of the PRP. Standardizing PRP preparation methods and selecting the most appropriate technique based on the specific clinical application is essential to ensuring consistent and predictable treatment outcomes [56].

Patient health and characteristics: The health status and individual characteristics of the patient are critical determinants of PRP composition. Patient-specific factors, such as age, genetics, and underlying medical conditions, can significantly influence the quality and effectiveness of PRP therapy. For instance, patients with chronic illnesses or those taking certain medications may exhibit variations in their blood components, affecting the platelet concentration and growth factor levels in the PRP. Healthcare providers must consider these patient-specific factors when tailoring PRP therapy to individual needs, ensuring the treatment is optimized for each patient [57].

Donor-to-donor variability: Even when following the same PRP preparation method, variations in PRP composition can arise from donor-specific factors. Baseline platelet counts and growth factor levels differ from one donor to another, contributing to these differences. Donor-to-donor variability adds complexity to PRP therapy, challenging predicting treatment outcomes with absolute certainty. Healthcare providers must acknowledge and account for this inherent variability when administering PRP therapy, emphasizing the need for a personalized and patient-specific approach [58].

Timing of blood collection: PRP composition may also be impacted by the time of blood collection in relation to the patient's most recent physical activity. Exercise or physical stress can transiently increase platelet counts in the bloodstream, potentially affecting the platelet concentration in the collected PRP. To mitigate this variability, it is advisable to establish guidelines for standardized patient preparation before PRP collection. Patients may be instructed to abstain from strenuous physical activity for a specified period before blood collection to minimize the influence of exercise-induced platelet changes on PRP quality [59].

Standardization challenges: The need for standardized protocols and guidelines for PRP preparation and administration poses a notable challenge. The absence of universally accepted standards can lead to variations in the composition of PRP preparations between different healthcare providers and clinical settings. Standardization challenges can result in inconsistencies in treatment responses and hinder meaningful comparisons between studies and clinical outcomes. Addressing these challenges requires collaborative efforts from the medical and scientific communities to establish clear and universally accepted protocols for PRP therapy, promoting more consistent and predictable treatment results [60].

Standardization and Regulation

Standardized PRP preparation protocols: Developing universally accepted protocols for platelet-rich plasma (PRP) preparation is imperative to ensure consistency and predictability in treatment outcomes. These standardized protocols should encompass several critical aspects, including the methods for blood collection, centrifugation techniques, and the handling of PRP. By adhering to standardized processes, healthcare providers can minimize the variability in PRP composition, which is essential for achieving more reliable and effective treatments. Standardization ensures that PRP preparations are consistent across healthcare facilities and providers, facilitating better comparisons of research findings and treatment outcomes. It also simplifies the training of medical personnel in the preparation process, reducing the risk of errors and deviations that could impact treatment efficacy [23].

Activation techniques: Clear guidelines for PRP activation methods are equally essential for optimizing the therapeutic effectiveness of PRP. Various activation techniques release platelet growth factors, such as calcium chloride, thrombin, or exogenous energy sources like ultrasound or laser. These activation methods should be rigorously evaluated and standardized to ensure the consistency of growth factor release and the safety of the process. Standardization in activation techniques helps to minimize the risk of over-activation or under-activation, which could affect treatment outcomes. Furthermore, it allows healthcare providers to select the most appropriate activation method based on the specific clinical application and desired release kinetics of growth factors, enhancing treatment customization and precision [22].

Quality control measures: Implementing rigorous quality control measures throughout the production of PRP is essential for ensuring the safety and efficacy of treatments. These measures encompass various facets of the PRP preparation process, including the monitoring of equipment, validation of PRP processing techniques, and sterilization verification. Regular equipment maintenance and calibration are critical to guaranteeing the accurate and consistent performance of centrifuges and other tools used in PRP preparation. Validation of PRP processing techniques involves verifying the reliability and reproducibility of the chosen protocols, reducing the risk of variability in PRP composition. Sterilization verification ensures that PRP preparations are free from contaminants that could lead to adverse patient reactions or infections. Robust quality control measures instill confidence in both healthcare providers and patients regarding the safety and reliability of PRP therapy, contributing to its responsible and effective use in clinical practice [61].

Regulatory oversight: Regulatory agencies are crucial in ensuring the safe and responsible use of platelet-rich plasma (PRP) therapy. Regulatory bodies must establish comprehensive PRP therapy guidelines and standards to achieve this. These regulations should encompass various aspects of the treatment process, including requirements for PRP preparation facilities, staff training, patient screening, and the monitoring of patient outcomes. Regulatory oversight provides a framework for responsible and ethical practices within the field. By defining the standards for facility infrastructure, staff competence, and patient selection criteria, regulatory agencies contribute to the safety and quality of PRP therapy. This oversight also helps protect patients from unscrupulous providers and substandard practices, ensuring that they receive treatment that adheres to established best practices and safety measures [61].

Patient safety: The primary priority in PRP therapy should always be patient safety. To prioritize patient well-being, guidelines should be created to determine patient eligibility for PRP therapy. This involves a thorough screening process to identify underlying health conditions, allergies, and contraindications that may affect the safety and efficacy of treatment. Additionally, continuous monitoring of patients during and after PRP therapy is crucial. This monitoring serves to detect and address any adverse reactions or complications promptly. By implementing these patient safety measures, healthcare providers can mitigate risks and provide the highest standard of care, ensuring patients receive PRP therapy safely and responsibly [23].

Data collection and reporting: Establishing standardized data collection and reporting practices is vital for systematically evaluating treatment outcomes and safety in PRP therapy. Collecting and reporting data consistently can build a substantial body of evidence for several critical purposes. It allows for evaluating treatment efficacy, safety, and patient outcomes, helping to refine best practices over time. Moreover, data collection and reporting contribute to the advancement of scientific knowledge in the field of regenerative medicine. This growing body of evidence benefits healthcare providers by enabling them to make data-driven decisions and improve treatment protocols, ultimately enhancing patient care. Additionally, it allows regulatory agencies to monitor the safety and effectiveness of PRP therapy more comprehensively, providing a basis for ongoing oversight and refinement of guidelines and standards [62].

Patient-Specific Factors Influencing Outcomes

Age and health status: Patient age and overall health status are pivotal factors that can influence tissues' regenerative potential and healing capacity in response to PRP therapy. Age-related changes in cellular activity, such as a decline in growth factor production and decreased cellular responsiveness, can affect the efficacy of PRP treatment. Individuals with compromised immune function or chronic health conditions may exhibit reduced regenerative capabilities. Patients with a robust immune system and good overall health tend to experience more robust responses to PRP therapy, as their bodies are better equipped to heal [63].

Genetic predispositions: An individual's reaction to PRP therapy is significantly influenced by their genetic predispositions. Genetic variations can influence the production of growth factors, the receptors for these factors, and the responsiveness of target tissues to bioactive molecules present in PRP. Understanding these genetic predispositions can provide valuable insights into patient-specific treatment responses. It enables healthcare providers to tailor PRP therapy approaches to maximize effectiveness and achieve more predictable outcomes. In the future, advances in personalized medicine may further enhance the customization of PRP treatment based on an individual's genetic profile [8].

Type and severity of wound or condition: Critical determinants of the therapeutic outcomes of PRP therapy are the type, location, and severity of the wound or medical condition being treated. The regenerative potential may be compromised in conditions with underlying infections, extensive tissue damage, or systemic inflammation. These factors can hinder the effectiveness of PRP treatment, as the body's resources are diverted toward addressing the underlying pathology. Therefore, healthcare providers need to assess the specific circumstances of each patient's condition to determine whether PRP therapy is an appropriate and beneficial intervention [64].

Lifestyle and environmental influences: Patient lifestyle choices, including diet, exercise, and exposure to environmental factors, can significantly impact the efficacy of PRP therapy. A healthy lifestyle that promotes overall well-being can complement the effects of PRP therapy. Regular exercise, a balanced diet, and avoiding harmful environmental factors, such as smoking or excessive alcohol consumption, can enhance the body's regenerative capabilities. Patients who adopt these healthy habits will likely experience more favorable treatment outcomes when undergoing PRP therapy [65].

Consideration of vasculopathic conditions: Examining potential contraindications for PRP usage is crucial, particularly in the context of common causes of vasculopathy. Conditions such as diabetes, cardiovascular disease, or autoimmune disorders can significantly influence the body's response to PRP therapy, potentially compromising tissue regeneration and impacting treatment efficacy. Additionally, it is pertinent to explore data related to vasospastic conditions like Raynaud's phenomenon. Understanding whether these conditions pose contraindications for PRP use is essential for healthcare providers. Moreover, specific medications may interact with PRP components, potentially altering treatment outcomes. Hence, a thorough assessment of comorbidities and the patient's medication profile is imperative to inform decisions about the suitability of PRP therapy and to optimize its benefits [66].

Cost Considerations and Accessibility

PRP preparation costs: The costs associated with PRP preparation are multifaceted. They encompass the expenses for acquiring and maintaining specialized equipment like centrifuges, essential for blood separation and PRP production. These machines must meet specific standards to ensure the quality of the PRP yield. Facilities must have the necessary infrastructure to maintain sterility during the preparation process. Additionally, skilled personnel are required to handle the equipment, collect blood samples, and oversee the entire preparation procedure. Choosing a particular PRP preparation method, such as double-spin centrifugation or buffy coat isolation, may also influence the costs, as some methods may necessitate more labor-intensive procedures or advanced equipment [23].

Activation method costs: The selection of an activation method in PRP therapy also bears associated costs. Various activation techniques, including calcium chloride, thrombin, or even exogenous energy sources like ultrasound or laser, have their own costs and requirements. These activation methods are crucial for releasing growth factors and bioactive molecules from platelets, which drive regenerative processes. The choice of activation method can affect the overall cost of PRP therapy and the release kinetics of growth factors, which may impact treatment efficacy and outcomes [22].

Medical personnel and facility fees: Beyond the costs directly tied to PRP preparation and activation, there are fees associated with the clinical administration of PRP therapy. These fees encompass the compensation of medical personnel involved in the patient's treatment, including nurses, physicians, and support staff. Facility fees pertain to using healthcare facilities and equipment for PRP administration. The complexity of the PRP therapy procedure, the level of medical expertise required, and the location of the treatment center can all influence the cost of these medical personnel and facility fees [67].

Health insurance coverage: The accessibility of PRP therapy can be significantly impacted by the availability of health insurance coverage. Unfortunately, not all health insurance plans cover PRP treatments, categorizing them as elective or experimental procedures. Consequently, patients may face the burden of high out-of-pocket expenses. The lack of insurance coverage for PRP treatments can limit access for individuals who would otherwise benefit from this regenerative therapy, placing financial strain on those seeking advanced wound healing and regenerative treatments [68].

Geographical disparities: Accessibility to PRP therapy can be notably uneven, with geographical disparities creating barriers to patient access. Patients in remote or underserved areas may struggle to access specialized PRP treatment centers. In urban centers, the availability of healthcare facilities and specialized clinics for PRP therapy is more common. However, individuals residing in rural or less densely populated regions may need help traveling to distant healthcare facilities. Geographical disparities can exacerbate healthcare inequities, as patients with limited access may miss out on the potential benefits of PRP therapy [69].

Research and development costs: Substantial expenses are involved in the research and development phase of PRP therapy. These costs include investments in laboratory research, clinical trials, and the validation of PRP therapy methods and applications. Research into the safety and efficacy of PRP treatment for various medical conditions and injuries demands significant funding. The rigorous testing and validation required to establish the clinical utility of PRP therapy can be time-consuming and financially intensive. While these costs contribute to the overall expenses associated with PRP therapy, they are instrumental in advancing the field and ensuring patient safety and treatment efficacy [70].

Patient affordability: The affordability of PRP therapy is paramount to making regenerative medicine accessible to a broad range of individuals. Treatment costs must align with patients' financial means to ensure that PRP therapy remains within reach for those seeking advanced wound healing and regenerative solutions. Patient affordability depends on factors such as insurance coverage, the pricing structure of PRP treatment, and the patient's financial situation. Efforts to control costs, increase transparency in pricing, and advocate for broader insurance coverage can help ensure that PRP therapy is financially accessible for patients across diverse socioeconomic backgrounds [53].

## Conclusions

In conclusion, the activation of PRP emerges as a pivotal factor in advancing skin wound healing. By harnessing the regenerative potential inherent in growth factors and bioactive molecules, activated PRP accelerates tissue repair, promotes angiogenesis, and enhances the overall wound-healing process. Throughout this comprehensive review, we have explored PRP's composition, mechanisms of action, clinical applications, and safety considerations. Notably, the potential of PRP extends beyond standalone therapy, prompting an investigation into combination therapies. Examples of such synergistic approaches warrant further exploration to maximize therapeutic efficacy. With its demonstrated ability to expedite healing rates and reduce complications, PRP stands poised to become an integral component of routine clinical practice, offering significant benefits to patients and healthcare systems. The future trajectory of PRP therapy will be influenced by ongoing research, technological advancements, the exploration of combination therapies, the paradigm of personalized medicine, and evolving regulatory standards. Collaborative efforts among researchers, healthcare providers, and regulatory bodies are poised to refine applications and safety protocols, setting new benchmarks in regenerative medicine and ultimately improving patient outcomes.
